# Prevalence of plasmid-mediated multidrug resistance determinants in fluoroquinolone-resistant bacteria isolated from sewage and surface water

**DOI:** 10.1007/s11356-016-6221-4

**Published:** 2016-02-19

**Authors:** Adriana Osińska, Monika Harnisz, Ewa Korzeniewska

**Affiliations:** Department of Environmental Microbiology, Faculty of Environmental Sciences, University of Warmia and Mazury in Olsztyn, Prawocheńskiego 1 Str., 10-720 Olsztyn, Poland

**Keywords:** Multidrug resistance, Fluoroquinolone, Tetracyclines, Beta-lactams, Wastewater, River water

## Abstract

Fluoroquinolones (FQs) are fully synthetic broad-spectrum antibacterial agents that are becoming increasingly popular in the treatment of clinical and veterinary infections. Being excreted during treatment, mostly as active compounds, their biological action is not limited to the therapeutic site, but it is moved further as resistance selection pressure into the environment. Water environment is an ideal medium for the aggregation and dissemination of antibiotics, antibiotic-resistant bacteria (ARB), and antibiotic resistance genes (ARGs), which can pose a serious threat to human health. Because of this, the aim of this study was to determine the number of fluoroquinolone-resistant bacteria (FQRB) and their share in total heterotrophic plate counts (HPC) in treated wastewater (TWW), and upstream and downstream river water (URW, DRW) samples where TWW is discharged. The spread of plasmid-mediated quinolone resistance (PMQR) determinants and the presence/absence of resistance genes to other most popular antibiotic groups (against tetracyclines and beta-lactams) in selected 116 multiresistant isolates were investigated. The share of FQRB in total HPC in all samples was rather small and ranged from 0.7 % in URW samples to 7.5 % in TWW. Bacteria from *Escherichia* (25.0 %), *Acinetobacter* (25.0 %), and *Aeromonas* (6.9 %) genera were predominant in the FQRB group. Fluoroquinolone resistance was mostly caused by the presence of the gene *aac*(*6*′)-*1b-cr* (91.4 %). More rarely reported was the occurrence of *qnr*S, *qnr*D, as well as *oqx*A, but *qnr*A, *qnr*B, *qep*A, and *oqx*B were extremely rarely or never noted in FQRB. The most prevalent bacterial genes connected with beta-lactams’ resistance in FQRB were *bla*_TEM_, *bla*_OXA_, and *bla*_CTX-M_. The *bla*_SHV_ was less common in the community of FQRB. The occurrence of *bla* genes was reported in almost 29.3 % of FQRB. The most abundant *tet* genes in FQRB were *tet*(A), *tet*(L), *tet*(K), and *tet*(S). The prevalence of *tet* genes was observed in 41.4 % of FQRB. The highest prevalence of multidrug-resistant (MDR) microorganisms was detected in TWW and DRW samples. It indicates that discharged TWW harbors multiresistant bacterial strains and that mobile PMQR and ARGs elements may have a selective pressure for species affiliated to bacteria in the river water.

**Electronic supplementary material:**

The online version of this article (doi:10.1007/s11356-016-6221-4) contains supplementary material, which is available to authorized users.

## Introduction

Antimicrobial resistance due to the continuous selective pressure from widespread use of antimicrobials in humans, animals, and agriculture has been a growing problem for decades (Baquero et al. 2008; Harnisz et al. 2015a; Kotlarska et al. 2015). Fluoroquinolones (FQs) are used extensively in both human and veterinary medicine, being considered important weapons against Gram-negative and Gram-positive organisms due to their selectively inhibiting bacterial DNA synthesis. They are the third largest group of antibiotics accounting for 17 % of the global market (Van Doorslaer et al. 2014). FQs are largely excreted as unchanged compounds in urine (45–62 %) and feces (15–25 %) and consequently discharged into hospital or municipal sewage (Kaplan et al. 2013). According to Kümmerer (2009), fluoroquinolones are not easily biodegradable and the mechanism for removing them from the environment may be associated with adsorption to sludge during biological treatment processes. Lindberg et al. (2006) and Fink et al. (2012) reported that FQs are not completely removed at wastewater treatment plants (WWTPs) and more than 70 % of ciprofloxacin and norfloxacin, antibiotics of the fluoroquinolones class, treated in biological treatment plants, remained adsorbed to sludge. Their study indicates that sludge is the main reservoir of fluoroquinolones and that these antibiotics are potentially released into the environment when biosolids are applied to agricultural areas. Some authors (Golet et al. 2002; Frade et al. 2014) have reported that fluoroquinolones become highly enriched in WWTPs’ effluents and sewage sludge in concentrations ranging from 0.3 to 1 μg/L and 1.4 to 31.2 mg per kg of dry matter, respectively. FQs are also strongly adsorbed to inorganic materials (Trivedi and Vasudevan 2007). Therefore, they can be rapidly transferred into the aquatic reservoirs and readily accumulate in their sediments (Pena et al. 2007; Sturini et al. 2012). Ciprofloxacin, the most frequently prescribed fluoroquinolone in Europe, has been classified as a poorly biodegradable antibiotic and detected at levels of hundreds of nanogram per liter in surface waters (Tello et al. 2012) and up to 5 μg/L in municipal WWTP’s effluents (Rosal et al. 2010). Due to a wide spectrum of FQs activity, their continuous introduction into the environment has qualitative and quantitative effects on the resident microbial community in the environment, consequently making environmental microbial communities the critical reservoirs for antibiotic resistance genes (Aminov 2009; Córdova-Kreylos and Scow 2007).

The low efficacy of sewage treatment may contribute to the dissemination of multidrug-resistant (MDR) bacteria from municipal sewage effluents directly into the water bodies (lakes/ rivers) (Finley et al. 2013; Korzeniewska and Harnisz 2013; Zheng et al. 2011). Indeed, wastewater has been recognized as a reservoir for ARGs, in which the presence of mobile genetic elements such as plasmids, insertion sequences, transposons and integrons has favored their dissemination. Thus, ubiquitous bacteria present in environmental samples may act as vectors for the dissemination of antimicrobial resistance (Harnisz et al. 2015b; Lupo et al. 2012; Stalder et al. 2012; Szczepanowski et al. 2009). Various genes encoding different resistance mechanisms on mobile genetic elements can decrease susceptibility to quinolone or fluoroquinolone antibiotics. These are often encoded on plasmids and known as plasmid-mediated quinolone resistance (PMQR) genes. The emergence of PMQR first reported in 1998 stimulated a great deal of interest in the transferable mechanism of quinolone resistance. So far, three different transferable quinolone resistance mechanisms have been identified: the *qnr* gene families (*qnr*A, *qnr*B, *qnr*S, *qnr*C, *qnr*VC, and *qnr*D) of proteins that protect DNA gyrase and topoisomerase IV from quinolones; *aac(6′)-Ib-cr* gene coding for aminoglycoside acetyltransferase that acetylate fluoroquinolones with a piperazinyl substituent, such as ciprofloxacin and norfloxacin; and *qep*A and *oqx*AB genes responsible for active efflux pumps (multidrug efflux pump) that confer decreased susceptibility to fluoroquinolones (Poirel et al. 2012; Ruiz et al. 2012). The relationship between PMQR and the use of other classes of antibiotics has been poorly investigated, and research has been conducted using mainly hospital strains. Hsu et al. (2010) demonstrated a relationship between quinolone resistance of clinical isolates and the use of quinolones, piperacillin/tazobactam, and carbapenems. Briales et al. (2012) also confirmed the association between quinolone resistance and resistance to other antimicrobial agents, particularly beta-lactams and aminoglycosides in isolates collected from hospital patients. Betitra et al. (2014) reported that PMQR genes are associated with the same mobile genetic elements as those of extended spectrum beta-lactamases (ESBL) genes in *Escherichia coli* causing urinary tract infection. The presence of mechanisms for broad-spectrum resistance on the same plasmid highlights the clinical importance of these genes and the potential for selection and dissemination of resistance to various antimicrobials through improper quinolone use.

The previous studies of the authors of this paper on the resistance to beta-lactams and tetracyclines indicated a growing insensitivity to these antibiotics among bacteria isolated from hospital and municipal sewage, as well as among environmental microorganisms (Harnisz et al. 2015b; Korzeniewska and Harnisz 2013; Korzeniewska et al. 2013). Furthermore, the results of these studies showed that the presence of multidrug resistance among the strains resistant to tetracycline has been associated with a risk of insensitivity to fluoroquinolones. The prevalence of tetracycline-insensitive bacteria isolated from wastewater and river water and parallel resistant to second generation fluoroquinolones ranged from 19 to 34 % (Harnisz 2013). Data from the European Antimicrobial Resistance Surveillance Network (EARS-Net) (ECDC (European Centre for Disease Prevention and Control) 2013) indicate a radical increase in fluoroquinolone resistance among Gram-negative bacteria in EU/EEA countries in the years 2010–2013. However, little is known about the prevalence of plasmid-mediated quinolone resistance determinants and the diversity of multidrug resistance in environmental isolates. Thus, the objective of this study was to determine the prevalence of fluoroquinolone-resistant bacteria in municipal wastewaters and surface water where the treated effluent of WWTP is discharged. Due to possible transmission of antibiotic-resistant microorganisms to the environment and possible transfer of their resistance genes to other bacteria in the sewage and water, the presence/absence of multiresistance genes in fluoroquinolone-resistant bacteria was analyzed in our study. In the present study, the number of FQRB in the total amount of bacteria isolated from wastewater and environmental samples was determined. An attempt was also made to identify the mechanisms responsible for multidrug resistance of these microorganisms and for its spreading among bacteria. Thus, the multiresistance of FQRB and the presence/absence of specific antibiotic resistance genes in selected FQRB were also studied.

The publication of study’s results could serve to the initiation of amendments to the existing legislation in Central and Eastern European countries concerning microbiological monitoring of sewage before its inflow into the surface water.

## Materials and methods

### Study sites and sampling

The WWTP’s process line comprises mechanical, biological, and chemical treatment sections and sludge processing units. The plant (located in Olsztyn, Poland) has the following technical parameters: treatment system-activated sludge; average processing capacity—60,000 m^3^/day; wastewater type—municipal wastewater; mechanical treatment devices—screenings; grit chamber and pre-sedimentation tank; biological treatment devices—separation chambers, aeration chambers, and secondary sedimentation tanks; and sedimentation devices—closed and open digestion chambers, belt filter press, and incinerator. Treated effluent is discharged to the Łyna River. The Łyna River is one of the largest watercourses in north-eastern Poland which is referred to as the Green Lungs of Poland. Gotkowska-Płachta et al. (2015) divided the river into two sections based on changes in water quality parameters: the upper, unpolluted section extending from headwaters to Olsztyn and the lower section with urban influences.

Samples of river water from river sections upstream and downstream of the wastewater discharge point and samples of treated wastewater were collected in winter (January), spring (April), summer (July), and autumn (October) 2014. Upstream river water (URW) and downstream river water (DRW) was sampled approximately 600 m away from the treated wastewater discharge point (TWW) (N = 53° 49′ 7.27″ and E = 20° 26′ 57.95″). In total, 4 URW, 4 TWW, and 4 DRW samples were collected in three measuring series during the study. Water and wastewater samples were collected into sterile bottles, transported to the laboratory at the temperature of 4 °C and processed on the day of collection.

### Physicochemical parameters

Physicochemical parameters of river water and treated wastewater samples, including temperature (°C), oxygen concentrations (mg/L), and pH, were determined using Hydrolab Multiprobe 12 (Scott).

### Heterotrophic plate counts and counts of fluoroquinolone-resistant bacteria

To obtain 30–300 colony forming units (CFU) per plate, TWW samples were diluted with saline water, and URW and DRW samples were passed through a cellulose filter (pore diameter 0.45 μm, Millipore) or diluted with saline water. Greater accuracy was achieved by plating triplicates. Heterotrophic plate counts (HPC) were determined on plates containing the tryptic soy agar (TSA, Oxoid). The plates were cultured at 30 °C for 24 h. The number of fluoroquinolone-resistant bacteria (FQRB) was determined on plates containing the TSA medium (Oxoid) with the addition of ciprofloxacin (2 mg/L) (Sigma). Antimicrobial dose was determined in accordance with “European Committee on Antimicrobial Susceptibility Testing; Breakpoints tables for interpretation of MICs and zones diameters” (EUCAST 2014). Resistant microorganisms were incubated at 30 °C for 24 h.

Cultured colonies of HPC and FQRB were counted, and the results were stated in terms of CFU per mL of river water or treated wastewater. ERIC (enterobacterial repetitive intergenic consensus sequence) PCR fingerprinting was applied to determine clonal relatedness of the isolates. The PCR reaction using ERIC1 and ERIC2 primers was done according to Versalovic et al. (1991). For further investigation, only the isolates without clonal relatedness were selected. A total of 116 colonies of FQRB were selected for further tests. They were purified on the TSA medium with ciprofloxacin (2 mg/L) and stored in tryptic soy broth (TSB, Oxoid) with glycerol at a temperature of −80 °C.

### Level of ciprofloxacin resistance and multiresistance of isolates

Minimum inhibitory concentrations (MICs) of ciprofloxacin were determined by the agar dilution method according to EUCAST guidelines (2014), with final antibiotic concentrations in the range of 1 to 512 μg/mL.

FQRB isolates were subjected to sensitivity tests against nine antimicrobials from five classes: (1) beta-lactams: amoxicillin/clavulanic acid (AMC 20/10 μg), mezlocillin (MEZ 75 μg), piperacillin (PRL 75 μg); ceftazidime (CAZ 30 μg), cefotaxime (CTX 30 μg); (2) glycylcyclines: tigecycline (TGC 15 μg); (3) aminoglycosides: tobramycin (TOB 10 μg); (4) trimethoprim/sulfamethoxazole (SXT 1.25/23.75 μg); and (5) tetracyclines: tetracycline (TE 30 μg). All disks were supplied by Oxoid. Resistance was estimated by measuring the growth inhibition zone according to guidelines (EUCAST 2014).

### Genomic and plasmid DNA extraction

Genomic and plasmid DNA was isolated in order to perform the amplification of 16S rRNA gene and antibiotic resistance genes, respectively. To extract genomic DNA, a loopful of FQRB bacterial colonies harvested from agar plates was suspended in 0.5 mL of sterile water, heated at 95 °C for 10 min, and centrifuged at 5000 rpm for 5 min at 4 °C. Plasmid DNA was extracted using Plasmid Mini Kits (A&A Biotechnology, Poland) according to the manufacturer’s instructions.

The concentration and quality of extracted DNA was determined by microspectrophotometry (NanoDrop® ND-1000, Willmington, DE). DNA was extracted in duplicate and stored at −20 °C for further analysis.

### Identification of FQRB and the occurrence of resistance genes

Resistant isolates were identified by 16S rRNA gene sequencing. Universal primers 27F and 1492R were used to amplify nearly full-length 16S rRNA gene sequences according to a previously described method (Gillan et al. 1998). After amplification, DNA was separated by electrophoresis in agarose gel stained with ethidium bromide (1 μg/mL). The exact 16S rRNA sequence was determined when the product was correct. PCR amplicons were sequenced using the ABI 3730xl automated fluorescent DNA sequencer (Applied Biosystems, Foster City, USA) and the BigDye® Terminator v3.1 Cycle Sequencing Kit (Applied Biosystems) in accordance with the manufacturer’s instructions. Identical primers for PCR and DNA sequencing were applied. DNA sequences were identified in the BLAST program available on the website of the National Center for Biotechnology Information (http://www.ncbi.nlm.nih.gov/BLAST).

The presence of 8 FQs resistance genes (*aac(6′)-Ib-cr*, *qnr*A, *qnr*B, *qnr*D, *qnr*S, *qep*A, *oqx*A, *oqx*B), 7 beta-lactam resistance genes (*bla*_CTX-M_, *bla*_CTX-M-1_, *bla*_CTX-M-2_, *bla*_CTX-M-9_, *bla*_SHV_, *bla*_TEM_, *bla*_OXA_), and 14 tetracycline resistance genes (*tet*(A), *tet*(B), *tet*(C), *tet*(D), *tet*(E), *tet*(G), *tet*(K), *tet*(L), *tet*(M), *tet*(O), *tet*(P), *tet*(S), *tet*(Q), *tet*(X)) was determined by standard PCR in plasmid DNA of all isolates. These genes were chosen due to the frequent resistance to beta-lactams and tetracyclines in FQRB. All primers had been previously validated (for primer sequences, amplicon sizes, annealing temperatures, references for each sequence, and additional details regarding PCR conditions, see Supplementary Material, Table S1).

In the presented study, the prevalence of genes responsible for resistance to fluoroquinolones was analyzed in all isolates, but determination of occurrence of beta-lactams and tetracycline resistance genes only in those isolates which were resistant to these antibiotics in phenotypic test.

Amplification products for each gene were purified with the GeneJET PCR Purification Kit (Thermo Fisher Scientific, Waltham, USA) and cloned into *E. coli* DH5α using the InsT/Aclone PCR Cloning Kit (Thermo Fisher Scientific, Waltham, USA). Plasmids carrying target genes were extracted and purified with the GeneJET Plasmid Miniprep Kit (Thermo Fisher Scientific, Waltham, USA). All plasmid extracts were screened for the presence of resistance genes. The formation of appropriately sized PCR products was evaluated by electrophoresis in agarose gels. Appropriately sized products were sequenced, and the resulting sequences were compared with GenBank sequences for the target genes using the BLAST alignment tool (http://www.ncbi.nlm.nih.gov/blast/).

Standard PCR mixtures without DNA template were used as negative controls in all reactions. Plasmids carrying resistance genes verified by sequencing were used as positive controls.

### Conjugation assay

Positive strains for the *qnr*, *qep*, *oqx*, *aac(6′)-Ib-cr*, *tet*, or *bla* genes (18 strains of the 3 most frequently isolated genera of bacteria: *Escherichia*, *Acinetobacter*, and *Aeromonas* from URW, TWW, and DRW samples) were examined for the ability to transfer resistance by conjugation (Korzeniewska and Harnisz 2013). The recipient strain was *E. coli* J53 (Rif^R^). Briefly, the donor and recipient strains were grown in LB medium (Merck) overnight at 30 °C, mixed in equal volumes, grown to mid-exponential phase (20 h), plated on Mueller–Hinton agar plates containing rifampicin (100 μg/mL) and ciprofloxacin (2 μg/mL), and incubated for 24 h at 30 °C. The transconjugants were tested for the presence of the transferred genes by PCR analyses. Plasmid DNA of donor strains and transconjugants was isolated with High Pure Plasmid Isolation Kit (Roche). All experiments were performed in triplicate. MIC values of ciprofloxacin, tetracycline, cefotaxime, and rifampicin of donor strains were determined according to the procedures mentioned above. The transfer frequency was calculated as a ratio of transconjugants to donor cells.

### Data analyses

Statistical analyses were carried out using the STATISTICA 10 software package (StatSoft Inc., 1984-2011). Because the data were not normally distributed, the Kruskal–Wallis test (KW), a non-parametric version of classical one-way analysis of variance ANOVA, was used to determine variations in the abundance of the studied bacterial groups and physicochemical parameters in samples from different sites. To detect the differences between each sampling site, the post hoc (p-h) pairwise multiple comparison of mean ranks was used. The correlations between physicochemical parameters and microbial counts were determined by Spearman’s rank correlation. The Wilcoxon test was used to check the magnitude of differences between resistance profiles and antimicrobial MICs in samples obtained from various sites (Stanisz 2007).

## Results and discussion

### Concentration of physicochemical parameters and counts of HPC and FQRB

The highest average temperature was observed in TWW samples at 15.6 ± 5.2 °C, and the lowest temperature was noted in URW samples at 11.5 ± 8.1 °C. URW samples showed the highest dissolved oxygen concentrations (9.4 ± 2.12 mg/L), whereas TWW samples contained the smallest amounts of dissolved oxygen (3.8 ± 1.35 mg/L). Similar trends were reported in respect of pH which was the highest in URW (7.8 ± 0.16) and the lowest in TWW samples (7.0 ± 0.19) (Table 1). Based on the evaluated physicochemical parameters, the quality of river water was described as satisfactory and it was consistent with the results reported by Gotkowska-Płachta et al. (2015) who studied the Łyna River in 2011–2012. The results of statistical analyses revealed significant variations in dissolved oxygen concentrations and pH of samples from different sites (KW, both *p* values <0.009). No significant differences in temperature were observed across the analyzed locations (KW, *p* > 0.5). The above could be attributed to the presence of a hydroelectric power plant on the Łyna River upstream from the URW sampling site which heated river water and leveled out its temperature across sampling sites (Harnisz 2013).

**Table 1:** Heterotrophic plate counts, counts of FQRB, and characteristics of water quality in URW, TWW, and DRW

Sampling sites	HPC, CFU/mL	FQRB, CFU/mL (% share of HPC)	Temperature, °C	Concentration of oxygen, mg/L	pH
URW	1650 ± 1110	12 ± 4 (0.72)	11.5 ± 8.1	9.4 ± 2.1	7.8 ± 0.16
TWW	41,300 ± 21,375	3100 ± 350 (7.5)	15.6 ± 5.2	3.8 ± 1.35	7.0 ± 0.19
DRW	5201 ± 2545	300 ± 40 (5.76)	12.3 ± 8.2	8.2 ± 2.2	7.6 ± 0.19

*URW* upstream river water, *TWW* treated wastewater, *DRW* downstream river water, *HPC* heterotrophic plate count, *FQRB* counts of fluoroquinolone-resistant bacteria

In the past few years, resistance to FQs has increased across the globe, thereby limiting available therapeutic options or resulting in treatment failure (Briales et al. 2012; Okade et al. 2014; Piekarska et al. 2015). The second-generation fluoroquinolone, ciprofloxacin, is widely used in human medicine in Poland (ECDC 2013), and it is probably the most abundant antibiotic compound detected in municipal biosolids due to its widespread use and sorption properties (Van Doorslaer et al. 2014; Xiong et al. 2015). Therefore, the aim of our study was to determine the number of FQRB and their share in total microbial populations inhabiting wastewater and other environmental compartments. The choice of antibiotic dose to culture total count of antibiotic-resistant bacteria is difficult because microbial communities in the environment comprise a wide variety of species of pathogenic, potentially pathogenic, and saprophytic bacteria. However, antibiotic MIC values are given only for specific bacteria as *E. coli*, *Pseudomonas aeruginosa*, etc. (EUCAST 2014). In our study, ciprofloxacin in dose 2 mg/L was chosen because this drug is used to treat infections caused by Gram-negative rods. These bacteria are wildly distributed in both wastewater and surface water.

The counts of bacteria resistant to ciprofloxacin are presented in Table 1 as collective data for all four sampling takings. The highest mean concentration of the FQRB reaching 3.1 × 10^3^ CFU/mL was noted in TWW samples, and the lowest reaching 12 CFU/mL was noted in URW samples. The differences in the abundance of antibiotic-resistant bacteria between sampling sites were confirmed by a statistical analysis (KW, all *p* values <0.013). The number of FQRB were statistically different between URW and TWW, TWW and DRW, as well as URW and DRW (p-h test, all *p* values <0.03). Bacteria resistant to FQs had the highest share of total HPC in TWW samples and the smallest share in URW samples. However, the share of FQRB in total HPC in all samples was small and ranged from 0.72 % in URW to 7.5 % in TWW samples. The differences in resistant bacteria’s share of total HPC at sampling sites were confirmed by statistical analyses (KW, all *p* values <0.02). Post hoc test used to determine differences in share of FQRB between sampling sites did not reveal statistical differences between TWW and DRW, which can imply that treated effluents affect their relative abundance in surface waters. Also results of Szczepanowski et al. (2009) indicate that the wastewater treatment process could be one of the routes leading to dissemination of antibiotic-resistant bacteria into the environment. In our study, a slightly lower share of FQRB in HPC isolated from DRW could be attributed to partial reduction of microorganisms’ number due to the dilution of treated sewage in river water. Similar relationships were observed by Korzeniewska and Harnisz (2013), who studied the prevalence of ESBL-positive *Enterobacteriaceae* in municipal sewage and their emission to the river water.

Bacterial counts were negatively correlated with dissolved oxygen concentrations and pH. Negative correlations between microbial populations, concentration of oxygen, and pH were obvious because treated wastewater was characterized by much higher bacterial concentrations but lower dissolved oxygen concentration (DOC) and pH levels in comparison with river water. Similar results were obtained by Harnisz (2013), who analyzed correlation between the concentration of physicochemical parameters and counts of tetracycline-resistant bacteria in river water and WWTP’s effluent.

### Level of ciprofloxacin resistance and multiresistance of isolates

**Fig. 1:**
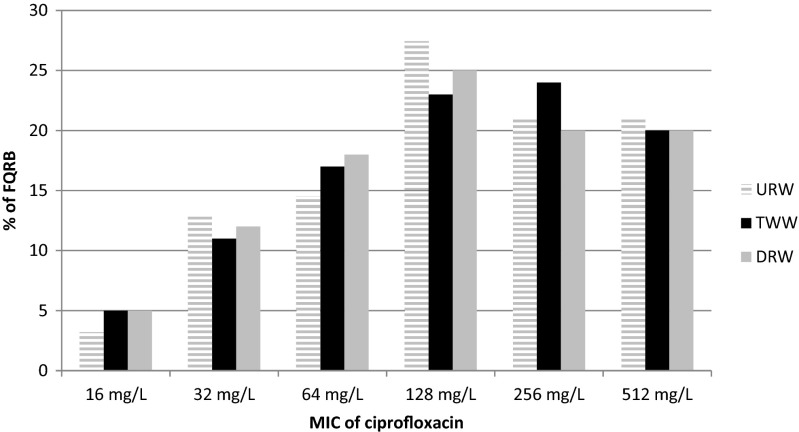
Minimum inhibitory concentration of ciprofloxacin in FQRB. *URW* upstream river water, *TWW* treated wastewater, *DRW* downstream river water

The minimum inhibitory concentrations of ciprofloxacin were very high and ranged from 16 to 512 mg/L (Table 2, Fig. 1). No significant differences in MIC values were observed between sampling sites (Wilcoxon test, *p* > 0.39). Rodríguez-Martínez et al. (2011) noted that isolates with PMQR have about 8–64-fold higher MIC of ciprofloxacin than isolates with other quinolone resistance.

**Table 2:** The level of resistance to ciprofloxacin, the pattern of multiresistance, and the occurrence of multidrug resistance determinants in selected fluoroquinolone-resistant bacteria

Isolates	Source	Determinants of quinolone resistance	Determinants of β-lactamase resistance	Determinants of tetracycline resistance	MIC of ciprofloxacin (mg/L)	Pattern of multiresistance
*E. coli*	TWW	*aac(6′)-1b-cr–oqx*B*–qnr*S	*bla*_CTX_ *–bla*_SHV_	*tet*(L)*–tet*(M)	64	AMC-SXT
		*aac(6′)-1b-cr*			64	CTX
		*qnr*D	*bla*_CTX_ *–bla*_OXA_ *–bla*_SHV_	*tet*(A)*–tet*(B)	128	MEZ-PRL-AMC-CAZ-CTX-SXT-TE-TOB
		*aac(6′)-1b-cr–oqx*A*-qnr*S			512	MEZ-PRL-AMC-CTX
		*aac(6′)-1b-cr–oqx*A*-qnr*S	*bla*_TEM_	*tet*(A)*–tet*(B)*–tet*(K)*–tet*(S)*–tet*(X)	512	MEZ-PRL-CTX-SXT-TE-TGC
		*qnrS*	*bla*_OXA_ *–bla*_TEM_	*tet*(A)*–tetA*(P)*–tet*(K)*–tet*(L)*–tet*(X)	256	MEZ-PRL-AMC-CTX-TE-TGC
		*aac(6′)-1b-cr–oqx*B		*tet*(K)*–tet*(L)*–tet*(X)	512	MEZ-PRL-CAZ-CTX-SXT-TE
		*aac(6′)-1b-cr–oqx*A*–ogx*B			128	MEZ-PRL-CAZ-CTX-SXT
		*aac(6′)-1b-cr–oqx*A*–qep*A	*bla*_SHV_		32	MEZ-PRL-AMC-CTX-TE
			*bla*_TEM_	*tet*(A)*–tet*(B)*–tet*(K)	32	MEZ-PRL-CTX-SXT-TE-TOB
		*aac(6′)-1b-cr–qnr*D		*tet*(A)*–tet*(S)	128	MEZ-PRL-CAZ-CTX-TE
		*aac(6′)-1b-cr*		*tet*(A)*–tet*(K)*–tet*(L)	64	MEZ-PRL-CAZ-CTX-SXT-TE
		*aac(6′)-1b-cr–qnr*D		*tet*(L)	64	MEZ-PRL-CAZ-CTX-TE-TOB-TGC
		*aac(6′)-1b-cr*		*tet*(M)	128	MEZ-PRL-CTX-SXT-TE-TGC
		*aac(6′)-1b-cr–qnr*S			256	CAZ-CTX-TE-TOB-TGC
		*aac(6′)-1b-cr*		*tet*(A)*–tet*(L)	256	MEZ-PRL-CTX-SXT-TE
		*qepA*	*bla*_TEM_	*tet*(A)*–tet*(L)	256	MEZ-PRL-AMC-CTX-SXT-TE
		*aac(6′)-1b-cr*			32	MEZ-PRL-CTX-TE-TOB
		*aac(6′)-1b-cr–oqx*A*–qep*A	*bla*_TEM_	*tet*(A)*–tet*(S)	32	MEZ-PRL-AMC-CTX-SXT-TE-TGC
	DRW	*aac(6′)-1b-cr–oqx*A*–qnr*S	*bla*_TEM_	*tet*(A)*–tet*(B)*–tet*(K)*–tet*(S)*–*_._ *tet*(X)	32	MEZ-PRL-AMC-CTX-SXT-TE
		*aac(6′)-1b-cr–qnr*S	*bla*_TEM_	*tet*(A)*–tet*(B)	64	MEZ-PRL-AMC-CTX-SXT-TE-TOB
		*aac(6′)-1b-cr–oqx*A*–qnr*S	*bla*_CTX_ *–bla*_TEM_	*tet*(A)*–tet*(B)	512	MEZ-PRL-AMC-CTX-SXT-TE
		*aac(6′)-1b-cr*	*bla*_TEM_	*tet*(B)	256	MEZ-PRL-CTX-SXT-TE-TOB
		*aac(6′)-1b-cr*			256	MEZ-PRL-CAZ-CTX-SXT-TE-TGC
		*aac(6′)-1b-cr–qnr*S	*bla*_OXA_ *–bla*_TEM_	*tet*(A)*–tet*(B)	128	MEZ-PRL-AMC-CAZ-CTX-SXT-TE-TOB-TGC
		*aac(6′)-1b-cr*	*bla*_TEM_	*tet*(B)	128	MEZ-PRL-CTX-SXT-TE
		*aac(6′)-1b-cr*		*tet*(K)*–tet*(L)*–tet*(M)	256	MEZ-PRL-CTX-TE
		*aac(6′)-1b-cr–qnr*D*–qnr*S			32	MEZ-PRL-CAZ-CTX-TE
		*aac(6′)-1b-cr–oqx*A*–qnr*S			512	MEZ-PRL-AMC-CAZ-CTX-TE-TGC
*Acinetobacter* sp.	URW	*aac(6′)-1b-cr*			32	MEZ-PRL-CTX
		*aac(6′)-1b-cr–qnr*S			512	MEZ-PRL-AMC-CAZ-CTX-SXT-TGC
		*aac(6′)-1b-cr–qnr*S			128	MEZ-PRL-CTX-SXT
		*aac(6′)-1b-cr–qnr*S	*bla*_OXA_		512	MEZ-PRL-CAZ-CTX-STX-TOB-TGC
		*aac(6′)-1b-cr–oqx*A*–qnr*D	*bla*_OXA_		128	MEZ-PRL-CAZ-CTX
	TWW	*qnr*D*–oqx*B			64	MEZ-PRL-CAZ-CTX
		*aac(6′)-1b-cr*	*bla*_OXA_	*tet*(A)*–tet*(X)	64	MEZ-PRL-AMC-CTX-TE-TOB-TGC
		*aac(6′)-1b-cr–qnr*D*–qnr*S	*bla*_CTX_	*tet*(A)*–tet*(K)*–tet*(L)	128	MEZ-PRL-CAZ-CTX-TE
		*aac(6′)-1b-cr–qnr*D*–qnr*S		*tet*(A)*–tet*(K)*–tet*(L)	256	MEZ-PRL-AMC-CAZ-CTX-TE-TGC
		*aac(6′)-1b-cr–oqx*A*–qnr*D	*bla*_OXA_		256	MEZ-PRL-CTX
		*aac(6′)-1b-cr–oqx*B			256	CAZ-CTX-TOB
	DRW	*aac(6′)-1b-cr–qnr*D*–qnr*S	*bla*_CTX_ *–bla*_OXA_		128	CTX
		*aac(6′)-1b-cr–qnrS*		*tet*(A)*–tet*(K)*–tet*(L)*–tet*(M)	64	CTX-SXT-TE-TOB
		*aac(6′)-1b-cr–oqx*A*-qnr*S	*bla*_CTX_		128	PRL-CAZ-CTX-SXT
		*aac(6′)-1b-cr–qnr*D		*tet*(K)*–tet*(L)*–tet*(S)	128	MEZ-PRL-AMC-CAZ-CTX-TE
		*aac(6′)-1b-cr*		*tet*(A)*–tet*(K)	256	MEZ-PRL-AMC-CAZ-CTX-SXT-TE
		*aac(6′)-1b-cr–oqx*A*–qnr*D		*tet*(L)*–tet*(S)	32	MEZ-PRL-CTX-SXT-TE
		*aac(6′)-1b-cr–oqx*A			32	MEZ-PRL-AMC-CAZ-CTX-SXT-TE-TGC
		*aac(6′)-1b-cr–qnr*D			16	MEZ-PRL-CAZ-CTX-SXT-TE-TGC
		*aac(6′)-1b-cr–oqx*A	*bla*_CTX_	*tet*(S)	512	MEZ-PRL-CAZ-CTX-SXT-TE-TOB-TGC
		*aac(6′)-1b-cr–oqx*A	*bla*_CTX_	*tet*(S)	512	MEZ-PRL-AMC-CAZ-CTX-SXT-TE-TGC
		*aac(6′)-1b-cr–qnr*D	*bla*_OXA_	*tet*(A)*–tet*(O)	128	PRL-CTX-TE-TGC
		*aac(6′)-1b-cr*		*tet*(K)*–tet*(L)	512	MEZ-PRL-AMC-CAZ-CTX-SXT-TE-TOB-TGC
		*aac(6′)-1b-cr–oqx*A*–qnr*D	*bla*_CTX_ *–bla*_TEM_		16	CTX
*Acinetobacter johnsonii*	URW	*qnr*D		*tet*(L)	512	MEZ-PRL-AMC-CAZ-CTX-SXT-TE-TGC
	TWW	*oqx*B*–qep*A*–qnr*S		*tet*(K)*–tet*(L)	64	MEZ-PRL-AMC-CAZ-CTX-SXT-TE
*Acinetobacter guillouiae*	URW	*aac(6′)-1b-cr–oqx*A		*tet*(K)*–tet*(L)*–tet*(S)	128	MEZ-PRL-AMC-CTX-SXT-TE-TOB-TGC
		*aac(6′)-1b-cr–oqx*A*–qnr*D		*tet*(L)*–tet*(S)	128	MEZ-PRL-CAZ-CTX-SXT-TE
*Acinetobacter bouvetii*	TWW	*aac(6′)-1b-cr–qep*A		*tet*(S)	512	MEZ-PRL-CAZ-CTX-SXT-TE-TGC
*Aeromonas* sp.	URW	*aac(6′)-1b-cr–oqx*A	*bla*_TEM_		512	MEZ-PRL-CAZ-CTX-SXT-TGC
		*aac(6′)-1b-cr*		*tet*(L)*–tet*(S)	256	MEZ-PRL-AMC-CAZ-CTX-SXT-TE
		*aac(6′)-1b-cr*		*tet*(K)*–tet*(M)	256	MEZ-PRL-CAZ-CTX-SXT-TE-TGC
	TWW	*aac(6′)-1b-cr–oqx*A*–qnr*S	*bla*_CTX_ *–bla*_OXA_	*tet*(E)*–tet*(K)*–tet*(L)*–tet*(S)	128	MEZ-PRL-AMC-CAZ-CTX-SXT-TE-TOB-TGC
	DRW	*aac(6′)-1b-cr*	*bla*_SHV_		128	MEZ-PRL-AMC-CTX-SXT-TOB
		*aac(6′)-1b-cr–oqx*A*–qnr*D			128	MEZ-PRL
		*aac(6′)-1b-cr–oqx*A*–qnr*D	*bla*_OXA_	*tet*(E)	128	MEZ-PRL-CTX-TE-TOB
*Aeromonas salmonicida*	DRW	*aac(6′)-1b-cr–oqx*A	*bla*_OXA_	*tet*(A)*–tet*(S)*–tet*(X)	128	MEZ-PRL-TE-TOB
*Bacillus* sp.	URW	*aac(6′)-1b-cr–qnr*D			16	MEZ-PRL-CTX-AMC-TE
		*aac(6′)-1b-cr–oqx*B			128	SXT
	TWW	*aac(6′)-1b-cr–qnr*D*–qnr*S		*tet*(A)	512	MEZ-PRL-CAZ-CTX-SXT-TE-TOB
		*oqx*A*–qnr*S			32	CAZ-TOB
	DRW	*aac(6′)-1b-cr–qnr*D			256	MEZ-PRL-AMC-CTX-SXT-TE
		*aac(6′)-1b-cr–qnr*S			64	CAZ-CTX
*Flavobacterium* sp.	URW	*aac(6′)-1b-cr*			256	TOB
		*aac(6′)-1b-cr*			256	MEZ-PRL-CAZ-CTX-SXT-TGC-TE
		*aac(6′)-1b-cr*			64	MEZ-PRL-CTX
	TWW	*aac(6′)-1b-cr*			128	MEZ-PRL-CTX-TOB
	DRW	*aac(6′)-1b-cr*			512	PRL-CTX-TOB-TGC-TE
*Klebsiella* sp.	TWW	*aac(6′)–1b-cr*			128	CTX
		*aac(6′)-1b-cr*			128	MEZ-PRL-CAZ-CTX-TGC
		*aac(6′)-1b-cr*			512	PRL-CAZ-CTX-SXT-TOB
	DRW	*aac(6′)-1b-cr*			64	PRL-CAZ-CTX-SXT-TOB
*Photobacterium* sp.	URW	*aac(6′)-1b-cr*			256	PRL-AMC-CAZ-CTX-SXT-TE-TGC
		*aac(6′)-1b-cr*			32	CAZ-TOB
	TWW	*aac(6′)-1b-cr*			128	MEZ-CTX-TE-TOB
	DRW	*aac(6′)-1b-cr*			64	CAZ-TOB
*Pseudomonas* sp.	URW	*aac(6′)-1b-cr–qnr*D			64	MEZ-AMC-CAZ-CTX-SXT
	TWW	*aac(6′)-1b-cr – oqx*B*–qnr*D			256	SXT
		*aac(6′)-1b-cr–qep*A	*bla*_SHV_	*tet*(A)*–tet*(K)	512	MEZ-AMC-CTX-SXT-TE-TGC
	DRW	*aac(6′)-1b-cr*			64	AMC-CAZ-CTX-SXT
*Cronobacter* sp.	URW	*aac(6′)-1b-cr*			128	MEZ-PRL-CAZ-CTX-SXT-TE
	TWW	*aac(6′)-1b-cr*			256	MEZ-PRL-CTX-SXT-TE
	DRW	*aac(6′)-1b-cr–qnr*D			256	MEZ-PRL-CAZ-CTX-TE-TGC
*Sphinogobacterium* sp.	URW	*aac(6′)-1b-cr–qnr*S		*tet*(E)*–tet*(K)*–tet*(S)	512	AMC-CTX-TE
	TWW	*aac(6′)-1b-cr*		*tet*(A)	256	CTX-SXT-TE
	DRW	*aac(6′)-1b-cr–oqxA*		*tet*(A)*–tetA*(P)*–tet*(B)*–tet*(M)*–tet*(X)	512	MEZ-PRL-CTX-TOB-TE
*Acidovorax* sp.	TWW	*aac(6′)-1b-cr–qep*A*–qnr*D	*bla*_CTX_		512	MEZ-PRL-AMC-CTX-CAZ-SXT-TE-TGC
	DRW	*aac(6′)-1b-cr*			32	
*Hydrogenophaga* sp.	TWW	*aac(6′)-1b-cr*			128	SXT-TE
	DRW	*qnr*D		*tet*(K)	512	MEZ-PRL-CAZ-CTX-TE
*Kurthia* sp.	TWW	*aac(6′)-1b-cr–qnr*S			256	MEZ-PRL-AMC-CAZ-CTX-SXT-TE-TGC
	DRW	*aac(6′)-1b-cr–qnr*D			64	SXT
*Lysinibacillus* sp.	TWW	*aac(6′)-1b-cr*			512	AMC-CTX
	DRW	*aac(6′)-1b-cr*			256	CAZ-CTX-TE-TOB
*Pedobacter* sp.	URW	*aac(6′)-1b-cr–qnr*S			64	AMC-CTX-TE
	DRW	*aac(6′)-1b-cr–oqx*A		*tet*(L)*–tet*(S)	256	MEZ-PRL-CTX-SXT-TE-TGC
*Providencia* sp.	TWW	*aac(6′)-1b-cr–qnr*D			512	PRL-CTX-CAZ-SXT-TE-TOB
	DRW	*aac(6′)-1b-cr*			64	CAZ-TOB
*Psychrobacter* sp.	URW	*aac(6′)-1b-cr*			32	MEZ-PRL-CTX-TE-TOB-TGC
	DRW	*aac(6′)-1b-cr–oqx*A*–qnr*D			16	
*Shigella* sp.	TWW	*aac(6′)-1b-cr–oqx*A*–qnr*D*–qnr*S	*bla*_TEM_		256	CAZ-TOB
	DRW	*aac(6′)-1b-cr–oqx*A	*bla*_TEM_		512	MEZ-PRL-CTX-SXT-TE-TOB
*Vibrio* sp.	TWW	*qnr*D			64	MEZ-PRL-CAZ-CTX-TE-TOB
	DRW	*aac(6′)-1b-cr*			256	MEZ-PRL-CAZ-CTX-TE-TOB
*Arthrobacter* sp.	TWW	*aac(6′)-1b-cr*			128	AMC-CAZ-CTX-TOB
*Morganella* sp.		*aac(6′)-1b-cr*			128	MEZ-AMC-CTX-TE
*Staphylococcus* sp.		*n6′)-1b-cr*			256

**Fig. 2:**
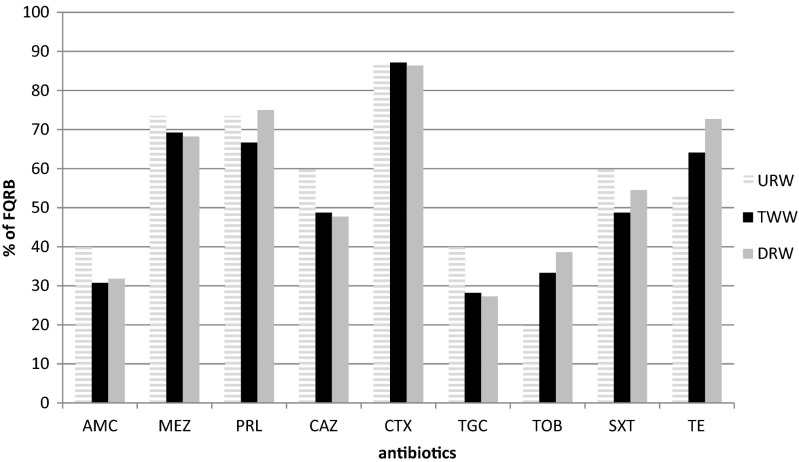
The frequency of drug resistance occurrence in FQRB. Antibiotics: amoxicillin/clavulanic acid (*AMC* 20/10 μg), mezlocillin (*MEZ* 75 μg), piperacillin (*PRL* 75 μg); ceftazidime (*CAZ* 30 μg), cefotaxime (*CTX* 30 μg); tigecycline (*TGC* 15 μg); tobramycin (*TOB* 10 μg); trimethoprim/sulfamethoxazole (*SXT* 1.25/23.75 μg), and tetracycline (*TE* 30 μg)

Analyses of the multidrug resistance profiles of 116 FQRB were also performed to receive a full characterization of drug-resistant isolates (Fig. 2). The results of the multidrug resistance profiles of 116 isolates are presented in Table 2. A comparison of the counts FQRB from different sampling sites revealed significant differences between URW and TWW as well as URW and DRW for tigecycline and tobramycin (ANOVA, all *p* values ≤0.03).

**Fig. 3:**
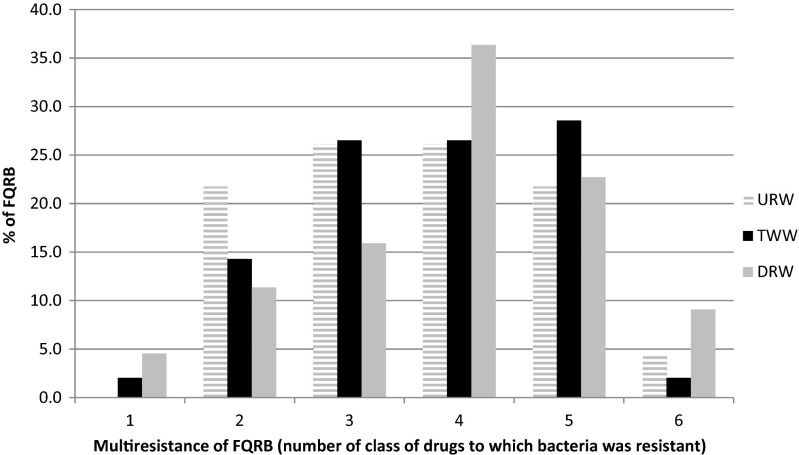
Multiresistance of FQRB

In the group fluoroquinolones-non-sensitive isolates, only 2 % strains from TWW and 4.5 % strains from DRW were resistant only to one antibiotic—ciprofloxacin. Other isolates were resistant to at least two classes of antibiotics (fluoroquinolones and drugs from other classes). The multiresistance was reported in 80, 82, and 83 % of isolates, from URW, TWW, and DRW, respectively. Most frequently reported was the presence of resistance to four classes of antibiotics in isolated strains (Fig. 3). The combined results of this experiment indicate that origin of isolates (URW, TWW, DRW) had no effect on the multidrug resistance of FQRB. The above is validated by the calculated percentage of multidrug resistance of FQ-resistant isolates in URW, TWW, and DRW (Fig. 3). The data indicate that isolates from TWW samples were characterized by similar multidrug resistance to isolates from URW and DRW. The presence of FQRB in URW suggests that ARGs can occur even in relatively pristine waters as the natural background level of antibiotic resistance in the environment (Marti et al. 2014).

### Identification of FQRB and determination of resistance genes

The results of 16S rRNA gene sequencing point to the predominance of Gram-negative bacteria from three bacterial genus: *Escherichia* (25.0 %, 29/116 isolates), *Acinetobacter* (25.0 %, 29/116 isolates), and *Aeromonas* (6.9 %, 8/116 isolates) in the group of FQRB (Table 2). The identified group of fluoroquinolone-resistant bacteria comprised also isolates representing *Acidovorax* sp., *Arthrobacter* sp., *Bacillus* sp., *Cronobacter* sp., *Flavobacterium* sp., *Hydrogenophaga* sp., *Klebsiella* sp., *Kurthia* sp., *Lysinibacillus* sp., *Morganella* sp., *Photobacterium* sp., *Pedobacter* sp., *Providencia* sp., *Pseudomonas* sp., *Psychrobacter* sp., *Shigella* sp., *Sphingobacterium* sp., *Staphylococcus* sp., and *Vibrio* sp. (Table 2). *E. coli* is one of the most common species identified among FQ-resistant clinical isolates (Briales et al. 2012; Guillard et al. 2014) and also among FQRB of animal and food origin (Yang et al. 2014). Marti et al. (2013) found that Gammaproteobacteria in TWW and upstream and downstream river sediment samples were mainly represented by the genera *Aeromonas* and *Acinetobacter*. Moreover, they also described members of these genera as multidrug-resistant microorganisms encoding resistance to beta-lactams, aminoglycosides, fluoroquinolones, and carbapenems.

ARGs have been becoming an increasing worldwide concern, as they pose a great threat to human health. Aquatic compartments, such as water and sediment, may be an ideal medium for the acquisition and dissemination of ARGs (Marti et al. 2014). The human activities were one of the most important factors influencing the distribution of ARGs in the aquatic environment.

**Fig. 4:**
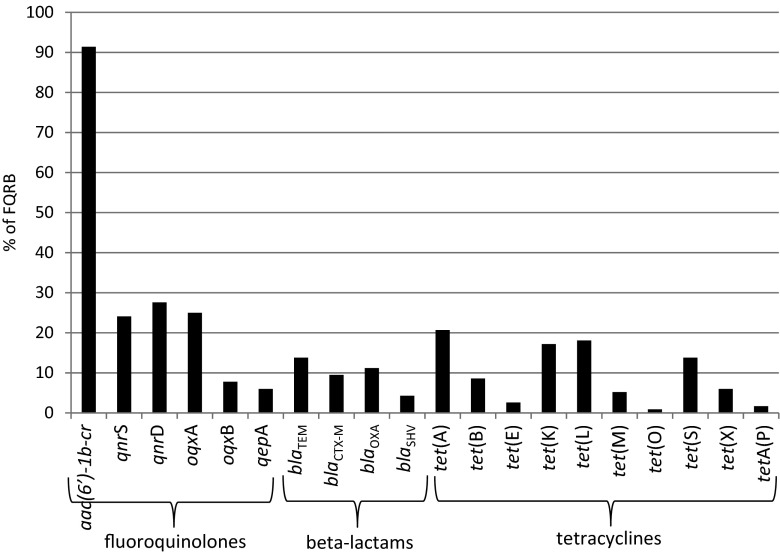
The prevalence of resistance genes in FQRB

Although fluoroquinolone resistance was originally thought to result from mutations in bacterial gyrase and topoisomerase IV genes, it is becoming apparent that it is also attributed to plasmid-associated resistance factors, which may propagate environmental antibiotic resistance (Kaplan et al. 2013). Fluoroquinolone resistance of FQRB was mostly caused by the presence of the PMQR determinants, gene *aac(6′)-1b-cr* (for *c*iprofloxacin *r*esistance) (Fig. 4), which is responsible for acetylate fluoroquinolones, kanamycin, tobramycin, and amikacin with a piperazinyl (Strahilevitz et al. 2009). In our study, the prevalence of *aac(6′)-1b-cr* gene was observed in more than 91 % (106/116 isolates) of FQRB. More rarely, the occurrence of *qnr*D, *oqx*A, and *qnr*S was reported, which was found in 27.6 % (32/116 isolates), 25.0 % (29/116 isolates), and 24.1 % (28/116 isolates) of FQRB, respectively. The *qnr*A, *qnr*B, *qep*A, and *oqx*B were extremely rarely or never noted in FQRB. Our results were similar to reports of Piekarska et al. (2015), who found that among PMQR determinants in ciprofloxacin-resistant clinical *Enterobacteriaceae*, *aac(6′)-Ib-cr* were predominant (85.7 %, 42/49 isolates). Guillard et al. (2014) found *aac(6′)-Ib-cr* in 96.7 % (118/122 isolates) of *E. coli* collected in hospitals located in the eastern region of France. Also Sana et al. (2014) reported that 97.0 % of uropathogenic *E. coli* harboring PMQR have encoded *aac(6′)-Ib-cr*. Additionally, most of these bacteria were harboring genes responsible for beta-lactams resistance. Other PMQR determinants, such as *qnr*A1, *qnr*A-like, *qnr*B1, and *qnr*S1, were detected less frequently. Additionally, no *qep*A gene was observed in their study. A similar predominance of *aac(6′)-Ib-cr* gene among PMQR determinants was also discovered by Briales et al. (2012) in clinical *E. coli* and *Klebsiella pneumoniae* producing ESBLs in Spain.

The most prevalent bacterial genes connected with beta-lactam resistance in FQRB were *bla*_TEM_, *bla*_OXA_, and *bla*_CTX-M_ and less often *bla*_SHV_. The TEM and SHV enzymes have been known for a long time; however, *bla*_SHV_ gene was generally chromosomally encoded (Bush 2010). In contrast to them, CTX-M beta-lactamases as a group have only increased in significance in recent years (Korzeniewska and Harnisz 2013; Li et al. 2015). The occurrence of at least one *bla* gene was reported in more than 30 % (35/116 isolates) of FQRB. The PMQR genes are usually associated with the same mobile genetic elements as those of ESBL genes. The presence of mechanisms for broad-spectrum resistance on the same plasmid highlights the importance of these genes and the potential for selection and dissemination of resistance to various antimicrobials through improper quinolone use (Betitra et al. 2014). Moreover, Vien et al. (2012) and Batard et al. (2013) observed that exposure to non-quinolone antibiotics, e.g., beta-lactams, can favor persistence of PMQRs and in consequence to decreased susceptibility to fluoroquinolones.

The most abundant *tet* genes in FQRB were *tet*(A), *tet*(K), *tet*(L), and *tet*(S). Genes *tet*(B), *tet*(E), *tet*(M), *tet*(O), *tet*(X), and *tetA*(P) were observed less frequently, but *tet*(C), *tet*(D), and *tet*(Q) were absent in FQRB (Fig. 4). Generally, the prevalence of at least one *tet* gene was observed in almost 42 % (48/116 isolates) of FQRB. It confirms the findings of other authors (Chopra and Roberts 2001) that over time bacteria have converted from carrying single *tet* genes to carrying multiple *tet* genes. The different *tet* genes can have either the same mode of action (efflux or ribosomal protection) or different modes of action (efflux and ribosomal protection). Moreover, the diversity of *tet* genes is also connected with their spread as a result of long-term use. Levy (1992) found that long-term use of tetracycline selects not only for tetracycline-resistant bacteria but also for multiple-drug-resistant species. Tetracycline resistance genes in both Gram-positive and Gram-negative species are often found on the same units (plasmids, transposons, or integrons) as other antibiotic resistance genes. There was no substantial difference in the prevalence of particular *tet* genes in FQRB isolated from URW, TWW, and DRW.

All of the studied *qnr*, *qep*, *oqx*, *aac(6′)-Ib-cr*, *tet*, and *bla* genes were transferable to *E. coli* J53 (Rif^R^) by conjugation assay, suggesting that, regardless of class, they were located on transferable elements. The frequency of conjugation ranged between 1.5 × 10^−6^ and 6.5 × 10^−5^ per donor strain (Supplementary Material, Table S2). This indicates a high possibility of horizontal gene transfer among strains of different genera within the sewage and environmental samples.

In the presented study, FQ-resistant *E. coli* were isolated only from TWW and DRW samples (Table 2). It can imply that the presence of these bacteria in the natural environment may be associated with the discharge of treated sewage. As frequently reported (Poirel et al. 2012), 40 % of isolates with PMQR mechanisms were MDR, encoding resistance to quinolones, beta-lactams, tetracycline, and/or sulphonamides. The association of the FQs’ resistance to MDR phenotypes was also observed for *E. coli* isolated from humans, animals, and the environmental samples by Chen et al. (2012) and for environmental microorganisms analyzed by Harnisz (2013). In the presented study, all (29 isolates) FQ-resistant *E. coli* isolated from TWW and DRW samples were also resistant to beta-lactams. Our results were similar to reports of Jones-Dias et al. (2013), who found that all *qnr*-producing *E. coli* recovered from food-producing animals in Portugal were non-susceptible to beta-lactam antibiotics, justified by the presence of beta-lactamases from TEM families. The other two predominant groups of identified FQRB (*Acinetobacter* sp. and *Aeromonas* sp.) were isolated from all three sampling sites, which confirms the prevalence of these genera in the natural as well as anthropogenically modified environment. Similar results are obtained by Harnisz et al. (2015b), who detected *Aeromonas* sp. and *Acinetobacter* sp. as the most frequent genera among oxytetracycline-resistant isolates in URW, TWW, and DRW.

## Conclusion

Multidrug mechanisms of resistance of FQRB and the interplay between different mechanisms of resistance have increased dramatically in recent years. The long-term fate of different strains of various species with diverse combinations of fluoroquinolone-resistance mutations is extremely hard to predict. Fluoroquinolone resistance is likely to be related to the biology of resistance as well as a direct response to drug pressure. Therefore, minimizing resistance will not be as simple as restricting the use of these agents. Currently, transferable plasmid-mediated quinolone resistance (PMQR) determinants in clinical isolates are extensively described around the world. They usually result in not only an increase in the MICs of quinolones but their presence may also facilitate the development of resistance to other antibiotic. Furthermore, their transmission to surface water through WWTP’s discharges is relatively easy. The emergence of new genes harboring multidrug resistance may continue in the next years, while the possible adaptation of enzymes, similar to what occurred with *aac(6′)-Ib*, may pose a potential risk. The current study demonstrated that both ARB and the coding genes of FQs’ antibiotic resistance are present in WWTP’s effluents. Moreover, genes encoding antibiotic resistance were shown to be transferrable to an *E. coli* recipient strain, which indicates a high possibility of horizontal gene transfer among strains of different genera within the sewage and environmental samples. In conclusion, this study demonstrated that despite the treatment, the municipal sewage may be a reservoir of antibiotic-resistant microorganisms and plasmid-mediated antibiotic resistance genes. In case of improper exploitation of WWTPs, the output water can contaminate other environmental sections, such as soil and water resources, and result in the emission of these contaminants. Urgent measures need to be taken to minimize the effects of releasing wastewaters into water resources.

## Electronic supplementary material

Below is the link to the electronic supplementary material.

ESM 1 (DOCX 24.9 kb)
