# Supplementary material for: Prevalence of plasmid-mediated multidrug resistance determinants in fluoroquinolone-resistant bacteria isolated from sewage and surface water
Source: Environ Sci Pollut Res Int. 2016 Feb 19;23:10818–31. doi: 10.1007/s11356-016-6221-4 (PMC4884563; doi:10.1007/s11356-016-6221-4)
Supplement: Supplementary file 1 — (DOCX 24.9 kb) [file 11356_2016_6221_MOESM1_ESM.docx]

**Prevalence and characterization of plasmid-mediated multi-drug resistance determinants in fluoroquinolone-resistance bacteria from sewage and surface water**

*Adriana Osińska,* *Monika Harnisz, Ewa Korzeniewska***,*

Department of Environmental Microbiology, Faculty of Environmental Sciences, University of Warmia and Mazury in Olsztyn, Prawocheńskiego 1 Str., 10-720 Olsztyn, Poland

*Corresponding Author Phone: +48 895233752; fax: +48 895234532; e-mail: ewa.korzeniewska@uwm.edu.pl

**Table S1: Qualitative and quantitative PCR (qPCR) primers and conditions used in this study**

| Target gene | Primer sequence (5’-3’) | Amplicon  size (bp) | Annealing  temperature in standard PCR  (°C) | Reference |
| --- | --- | --- | --- | --- |
| *16S rRNA* | CGGTGAATACGTTCYCGG | 1465 | 56 | Gillan et al. (1998) |
|  | GGWTACCTTGTTACGACTT |  |  |  |
| *aac(6’)-Ib-cr* | TTGCGATGCTCTATGAGTGGCTA | 482 | 55 | Park et al. (2006) |
|  | CTCGAATGCCTGGCGTGTTT |  |  |  |
| *qnrA* | ATTTCTCACGCCAGGATTTG | 516 | 53 | Robicsek et al. (2006) |
|  | GATCGGCAAAGGTTAGGTCA |  |  |  |
| *qnrB* | GGMATHGAAATTCGCCACTG | 264 | 54 | Cattoir et al. (2007) |
|  | TTTGCYGYYCGCCAGTCGAA |  |  |  |
| *qnrD* | CGAGATCAATTTACGGGGAATA | 465 | 50 | Li et al. (2012) |
|  | AACAAGCTGAAGCGCCTG |  |  |  |
| *qnrS* | GCAAGTTCATTGAACAGGGT | 428 | 54 |  |
|  | TCTAAACCGTCGAGTTCGGCG |  |  |  |
| *qepA* | CCAGCTCGGCAACTTGATAC | 570 | 60 |  |
|  | ATGCTCGCCTTCCAGAAAA |  |  |  |
| *oqxA* | CTCGGCGCGATGATGCT | 392 | 57 |  |
|  | CCACTCTTCACGGGAGACGA |  |  |  |
| *oqxB* | TCCTGATCTCCATTAACGCCCA | 131 | 64 |  |
|  | ACCGGAACCCATCTCGATGC |  |  |  |
| *bla*_CTX-M_ | SCSATGTGCAGYACCAGTAA | 544 | 55 | Saladin et al. (2002) |
|  | CCGCRATATGRTTGGTGGTG |  |  |  |
| *bla*_CTX-M-1_ | GGTTAAAAAATCACTGCGTC | 864 | 55 |  |
|  | TTGGTGACGATTTTAGCCGC |  |  |  |
| *bla*_CTX-M-2_ | ATGATGACTCAGAGCATTCG | 866 | 55 |  |
|  | TGGGTTACGATTTTCGCCGC |  |  |  |
| *bla*_CTX-M-9_ | ATGGTGACAAAGAGAGTGCA | 864 | 55 |  |
|  | CCCTTCGGCGATGATTCTC |  |  |  |
| *bla_SHV_* | GATGAACGCTTTCCCATGATG | 214 | 61 | Kim et al. (2009) |
|  | CGCTGTTATCGCTCATGGTAA |  |  |  |
| *bla_TEM_* | AGTGCTGCCATAACCATGAGTG | 431 | 61 |  |
|  | CTGACTCCCCGTCGTGTAGATA |  |  |  |
| *bla_OXA_* | ATTATCTACAGCAGCGCCAGTG | 296 | 61 |  |
|  | TGCATCCACGTCTTTGGTG |  |  |  |
| *tet(A)* | GCTACATCCTGCTTGCCTTC | 210 | 55 | Ng et al. (2001) |
|  | CATAGATCGCCGTGAAGAGG |  |  |  |
| *tet(B)* | TTGGTTAGGGGCAAGTTTTG | 659 | 55 |  |
|  | GTAATGGGCCAATAACACCG |  |  |  |
| *tet(C)* | CTTGAGAGCCTTCAACCCAG | 418 | 55 |  |
|  | ATGGTCGTCATCTACCTGCC |  |  |  |
| *tet(D)* | AAACCATTACGGCATTCTGC | 787 | 55 |  |
|  | GACCGGATACACCATCCATC |  |  |  |
| *tet(E)* | AAACCACATCCTCCATACGC | 278 | 55 |  |
|  | AAATAGGCCACAACCGTCAG |  |  |  |
| *tet(G)* | GCTCGGTGGTATCTCTGCTC | 468 | 55 |  |
|  | AGCAACAGAATCGGGAACAC |  |  |  |
| *tet(K)* | TCGATAGGAACAGCAGTA | 169 | 55 |  |
|  | CAGCAGATCCTACTCCTT |  |  |  |
| *tet(L)* | TCGTTAGCGTGCTGTCATTC | 267 | 55 |  |
|  | GTATCCCACCAATGTAGCCG |  |  |  |
| *tet(M)* | GTGGACAAAGGTACAACGAG | 406 | 55 |  |
|  | CGGTAAAGTTCGTCACACAC |  |  |  |
| *tet(O)* | AACTTAGGCATTCTGGCTCAC | 515 | 55 |  |
|  | TCCCACTGTTCCATATCGTCA |  |  |  |
| *tet(P)* | CTTGGATTGCGGAAGAAGAG | 676 | 55 |  |
|  | ATATGCCCATTTAACCACGC |  |  |  |
| *tet(S)* | CATAGACAAGCCGTTGACC | 667 | 55 |  |
|  | ATGTTTTTGGAACGCCAGAG |  |  |  |
| *tet(Q)* | TTATACTTCCTCCGGCATCG | 904 | 55 |  |
|  | ATCGGTTCGAGAATGTCCAC |  |  |  |
| *tet(X)* | CAATAATTGGTGGTGGACCC | 468 | 55 |  |
|  | TTCTTACCTTGGACATCCCG |  |  |  |
| ERIC | ATGTAAGCTCCTGGGGATTCAC | 50-4200 | 52/65 | Versalovic  (1991) |
|  | AAGTAAGTGACTGGGGTGAGCG |  |  |  |

**References**

Cattoir V., Laurent Poirel L., Rotimi V., Soussy C. J., Nordmann P., 2007. Multiplex PCR for detection of plasmid-mediated quinolone resistance qnr genes in ESBL-producing enterobacterial isolates. Journal of Antimicrobial Chemotherapy 60, 394–397

Gillan DC, Speksnijder A, Zwart G, De Ridder C. Genetic diversity of the biofilm covering Montacuta ferruginosa (Mollusca, bivalvia) as evaluated by denaturing gradient gel electrophoresis analysis and cloning of PCR-Amplified gene fragments coding for 16S rRNA. Applied and Environmental Microbiology 1998; 64: 3464-3472.

Kim J, Jeon S, Rhie H, Lee B, Park M, Lee H, Lee J, Kim S. (2009) Rapid Detection of Extended Spectrum β-Lactamase (ESBL) for *Enterobacteriaceae* by use of Multiplex PCR-based Method. Infection and Chemotherapy, 41(3):181-184.

Li J., Wang T., Shao B., Shen J., Wang S., Wu Y., 2012. Plasmid-Mediated Quinolone Resistance Genes and Antibiotic Residues in Wastewater and Soil Adjacent to Swine Feedlots: Potential Transfer to Agricultural Lands. Environmental Health Perspectives 120(8): 1144-1149

Ng, L.K., Martin, I., Alfa, M., and Mulvey, M. (2001) Multiplex PCR for the detection of tetracycline resistant genes. Molecular and Cellular Probes 15: 209-215.

Park, C.H., Robicsek, A., Jacoby, G.A., Sahm, D., Hooper, D.C., 2006. Prevalence in the United States of aac(6′)-Ib-cr encoding a ciprofloxacin-modifying enzyme. Antimicrobial Agents and Chemotherapy 50, 3953-3955.

Robicsek A., Strahilevitz J., Sahm D. F., Jacoby G. A., Hooper D. C., 2006. *qnr* Prevalence in Ceftazidime-Resistant *Enterobacteriaceae* Isolates from the United States. *Antimicrob. Agents Chemother.*  50(8): 2872- 2874

Saladin M., Cao V.T.B., Lambert T., Donay J.-L., Herrmann J.-L., Ould-Hocine Z., Verdet C., Delisle F., Philippon A., Arlet G., 2002. Diversity of CTX-M L-lactamases and their promoter regions from *Enterobacteriaceae* isolated in three Parisian hospitals. FEMS Microbiology Letters 209: 161-168.

Versalovic J, Koeuth T, Lupski JR (1991) Distribution of repetitive DNA sequences in eubacteria and application to fingerprinting of bacterial genomes. Nucleic Acids Res 19:6823–6831.

**Table S2. Mobility of antibiotic resistance genes**

| **Recipient** | **Donor** | **Resistance**  **genes** | **Frequency of transfer**  **(per donor cell)** |
| --- | --- | --- | --- |
| *E. coli* J53 (Rif^R^) | *Escherichia coli* | *aac(6’)-1b-cr* | 6.5x10^−5^ |
|  |  | *qepA* | 1.6x10^−6^ |
|  |  | *qnr*D | 3.2 × 10^−5^ |
|  |  | *qnrS* | 2.6 × 10^−5^ |
|  |  | *bla*_TEM_ | 4.7 × 10^−5^ |
|  |  | *bla*_SHV_ | 2.1 × 10^−5^ |
|  |  | *tet*(L) | 1.8 × 10^−5^ |
|  |  | *tet*(B) | 1.6 × 10^−5^ |
|  |  | *tet*(M) | 0.8 × 10^−5^ |
|  | *Acinetobacter* sp. | *qnr*D | 1.9 × 10^−5^ |
|  |  | *tet*(L) | 1.6 × 10^−5^ |
|  |  | *tet*(S) | 1.7 × 10^−5^ |
|  |  | *bla*_OXA_ | 1.8 × 10^−5^ |
|  |  | *bla*_CTX_ | 2.2 × 10^−5^ |
|  | *Aeromonas* sp. | *aac(6’)-1b-cr* | 4.8 × 10^−5^ |
|  |  | *tet*(E) | 1.5 × 10^− 6^ |
|  |  | *bla*_TEM_ | 3.4 × 10^−5^ |
|  |  | *bla*_SHV_ | 2.2 × 10^−5^ |
